# Understanding the Sociocognitive Process of Construction Workers’ Unsafe Behaviors: An Agent-Based Modeling Approach

**DOI:** 10.3390/ijerph17051588

**Published:** 2020-03-01

**Authors:** Gui Ye, Hongzhe Yue, Jingjing Yang, Hongyang Li, Qingting Xiang, Yuan Fu, Can Cui

**Affiliations:** 1 School of Management Science and Real Estate, Chongqing University; Chongqing 400045, China; 20180313018t@cqu.edu.cn (H.Y.); yangjingjing@cqu.edu.cn (J.Y.); xiangqingting@yeah.net (Q.X.); 201703021076@cqu.edu.cn (Y.F.); cuicanz@live.com (C.C.); 2 The International Research Center for Sustainable Built Environment, Chongqing University, Chongqing 400045, China; 3 Modern project management research centre, Chongqing University, Chongqing 400045, China; 4 School of Civil Engineering and Transportation, South China University of Technology, Guangzhou 510641, China; li.terryhy@yahoo.com; 5 State Key Laboratory of Subtropical Building Science, South China University of Technology, Guangzhou 510641, China

**Keywords:** construction worker, unsafe behaviors, sociocognitive process, social organizational factors, social groups, social interaction, agent-based modeling

## Abstract

Previous literature has recognized that workers’ unsafe behavior is the combined result of both isolated individual cognitive processes and their interaction with others. Based on the consideration of both individual cognitive factors and social organizational factors, this paper aims to develop an Agent-Based Modeling (ABM) approach to explore construction workers’ sociocognitive processes under the interaction with managers, coworkers, and foremen. The developed model is applied to explore the causes of cognitive failure of construction workers and the influence of social groups and social organizational factors on the workers’ unsafe behavior. The results indicate that (1) workers’ unsafe behaviors are gradually reduced with the interaction with managers, foremen, and workers; (2) the foreman is most influential in reducing workers’ unsafe behaviors, and their demonstration role can hardly be ignored; (3) the failure of sociocognitive process of construction workers is affected by many factors, and cognitive process errors could be corrected under social norms; and (4) among various social organizational factors, social identity has the most obvious effect on reducing workers’ unsafe behaviors, and preventive measures are more effective than reactive measures in reducing workers’ unsafe behaviors.

## 1. Introduction

Due to the inherent dangers of construction industry, the ever-changing site environment, and the lack of trained construction workers, the accident rate has always been at the forefront [1,2]. In the U.S., 1008 workers were killed on the job in the construction industry in 2018, accounting for 19% of total mortality and was far higher than other industries [3]. In the U.K., the construction industry contributes to 5% of the nation’s employment [4] but accounts for 20.41% of reported fatal injuries [5]. In China, there were 1752 deaths in the construction industry in the first half of 2018 [6]. It can be seen that safety incidents in the construction industry are still frequent and notorious and need to be addressed urgently. Accident investigations have demonstrated that construction workers’ unsafe behaviors are the primary and main cause of accidents [7,8]. Heninrich et al. [9] proposed that among 75,000 accidents cases, 88% of all industry accidents were caused by human errors. Suraji et al. [10] analyzed 500 accident records and found that 80% of accidents were caused by inappropriate construction operation. Fang and Wu [11] found that about one-third of construction workers did not behave safely. Previous efforts to reduce workers’ unsafe behavior have mainly relied on direct intervention such as rewards, punishments, behavior feedback, communication, and training [12,13]. These approaches, however, may not be effective in reducing workers’ unsafe behaviors because they are often based on the outcome of the unsafe behaviors, while ignoring the exploration of workers’ cognitive factors [1,14]. Cognitive psychology shows that behavior is a kind of product of human cognition. If workers perform unsafe behaviors, it must be a problem in the cognitive process of workers [15]. Therefore, it is necessary to explore the cognitive mechanism of workers to better control the unsafe behavior of workers.

The cognitive process of workers’ unsafe behaviors has become one of the core issues of safety studies in construction sites [16,17]. Many scholars have analyzed the unsafe behavior of workers from a cognitive perspective. Rasmussen’s ladder model, Wickens’ information processing model, and the IDAC (Information, Decision, and Action in Crew context) model are all individual cognitive models widely used to study workers’ unsafe behavior [17,18,19]. Kines [16] conducted a semistructured interview with construction workers injured by falling from heights. His research identifies a relationship between workers’ individual cognition and behaviors. Zhang and Fang [15] discussed the reasons why Chinese scaffolding workers do not use safety harness from the perspective of cognitive process; however, they only consider the influence of group factors on unsafe behavior from one stage of the cognitive process. Wang et al. [20] identified the key constraints that induce unsafe behaviors in complex work environments using a cognitive work analysis (CWA) approach, but CWA does not address organization-level or company-level factors. All of these cognitive process analysis make a great contribution to the exploration of worker’s unsafe behavior, but less consider human behavior at the organizational and group level. 

In fact, due to the complexity of both the physical and social nature of the construction workers’ working environment, workers’ unsafe behavior is not only the combined result of isolated individual cognitive processes, as well as the worker’s interaction with others. A large body of literature shows that social groups (coworkers, foremen, managers) can affect workers’ attitudes and unsafe behaviors [21,22,23]. Some studies have demonstrated the important role of coworker influence, foreman influence, manager feedback on workers’ unsafe behavior [24,25]. But few consider the impact of these three subjects on workers’ unsafe behaviors simultaneously. There is evidence that managers, foremen, coworkers, and workers interact with each other [23,26], and the relationship under interaction needs to be considered. Most of the existing literature analyzes the influence of foremen on the unsafe behavior of workers from the perspective of the supervision and safety management role [27,28]. While foremen were safety role models for their crew, if foremen overlook some safety issues, workers will have more excuses to ignore them [27]. Especially in China, the foreman culture is popular. The foreman and the worker are often connected by geographical factors. The relationship between them is not only the relationship between the manager and the managed person, but also the fellow of the township, relatives, and friends. As a special group of the construction industry, the influence of the foreman on the workers has a dual role of management and demonstration and should be considered. In addition, social groups often influence workers’ unsafe behaviors through social organizational factors, such as safety training, behavior feedback, and so on. Most previous literature has directly studied the impact of social organizational factors on workers’ unsafe behaviors based on macro-level correlation, statistical analysis, and empirical interpretation [26,29]. When social organizational factors change, it is difficult to predict how safety behavior will change. One possible reason is that the influence mechanism of social organization factors on workers’ unsafe behavior was not explored. Cognitive processes are the psychological mechanism of construction workers’ unsafe behavior. If social organizational factors are combined with construction workers’ cognitive processes, it can better explain the changes in construction workers’ unsafe behavior.

A failure in any stage of the cognitive processes would lead to an unsafe behavior [30]. Social organizational factors interact with cognitive processes and then cause unsafe behavior. Workers’ cognitive errors at one stage may be corrected under social organizational factors or social groups [21]. For example, the worker did not find the danger in the obtaining information stage, but the foreman told him/her that it was unsafe to do so; then, the worker was aware of the danger and finally took safe actions. His/her cognitive bias has actually been corrected. Workers’ sociocognitive processes that combine social organizational factors with cognitive processes may be complex, dynamic, nonlinear, and correctable [1,21,31], requiring reasonable methods to measure. Previous research methods mainly adopt a top-down approach, starting from the relationship suggested by theory and narrowing down into specific hypothesis tests. Although the methods enable the exploration of the relationships between sociocognitive processes and behaviors, it is important to note that such methods do not confirm individual differences and interactions among workers, foremen, coworkers, and managers [28]. 

Therefore, the research gaps can be summarized as including the following: first, the previous literature rarely considers the influence of managers, foremen, and coworkers on workers’ unsafe behaviors at the same time. The dual role of foreman’s management role and demonstration role is rarely considered. Second, the mechanism of the influence of social organization factors on workers’ unsafe behaviors is still unclear, and it is difficult to understand the influence of social organizational factors on workers’ unsafe behaviors. Third, workers’ cognitive process failures may be corrected by social organization factors, which have not been specified in previous studies. Fourth, workers’ sociocognitive process is affected by a variety of factors and needs appropriate measures.

To fill these gaps, this paper proposes an agent-based modeling (ABM) approach to study a complex system of unsafe behavior comprised of cognitive processes, individual cognitive factors, social organizational factors, and social groups, and then gives reasonable suggestions to reduce unsafe behavior. As a bottom-up method, ABM is more suitable for modeling inherently nonlinear [32], dynamic [33], multi-factor, computational, and flexible [34] models, and better to understand the sociocognitive processes of workers. In this paper, the individual cognitive factors and social organizational factors that affect construction workers’ unsafe behavior are first identified through empirical evidence and theoretical foundations. Workers’ sociocognitive model is then constructed, and the ABM method is used to investigate (1) how workers’ unsafe behaviors and risk acceptance change under the interaction of three social groups: managers, foremen, and coworkers; (2) what the dynamic factors of cognitive process failure of workers’ unsafe behavior are and whether they can be corrected; (3) how sociocognitive process of workers’ unsafe behaviors change under the different sociocognitive factors. The proposed framework has the potential to analyze the sociocognitive process of construction workers and to assess the impact of multiple management measures on unsafe behavior before implementation, thus yielding theoretical and practical implications for reducing occupational injuries and deaths among construction workers. 

## 2. Literature Review

### 2.1. Sociocognitive Process of Construction Workers

#### 2.1.1. Individual Cognitive Process of Construction Workers

Cognitive psychology studies human perception, attention, memory, mental (i.e., appearance), language, and thinking/decision making [35]. From the perspective of information processing, cognitive processes include receiving, storing, and using information as three stages [35]. According to the information processing theory, workers’ cognitive process can be divided into three stages: obtaining information, understanding information, and responding and taking action. The obtaining information stage represents workers’ searching and detecting of potential hazards in the outside environment [31]. In the next phase, workers form a certain cognitive response and estimate the hazard, which can translate the hazard information into problems or goals [31]. In the end, workers take the hazard evaluation as a judgment of whether to take unsafe behavior and select a certain response. Each stage can be broken down until the problem is solved [1,17]. Thus, workers’ cognitive processes of unsafe behaviors are nonlinear. Meanwhile, the cognitive process is a dynamic process which is influenced by some dynamic psychological factors. For example, the interaction between mental state and the cognitive process activity is a dynamic process of mutual influences [17]. Dynamic influencing factors of thinking can better explain the nonlinear and dynamic characteristics of cognitive processes.

In the stage of obtaining information, safety awareness, experience, and safety knowledge will affect the workers’ search of potential hazards. If workers have limited actual hazard information on the site, they may not expect hazards existing nearby. Thus, they have insufficient consciousness for the search for danger or cannot find risks in time when searching unconsciously, which may result in cognitive errors [31]. In addition, experience will affect workers’ perception of risk [36]. Abundant working experience makes workers more conversant in potential risks regarding projects [37]. Safety knowledge refers to the ability and skills to comprehend, master, and carry out related rules and regulations [38]. With a low level of safety knowledge, an inexperienced worker may not have the ability to detect a surrounding hazard and to recognize the risk [30].

In the understanding information stage, construction workers predict and assess the hazards when conducting operations onsite. This process requires an adequate safety knowledge and attitude. If workers’ safety knowledge is not enough, they cannot fully understand the risks. It is important to increase a worker’s knowledge of associated hazards and how to avoid them. Attitude refers to “the degree to which a person has a favorable or unfavorable evaluation or appraisal of the behavior in question” [38]. If workers tend to be risk averse, they often overestimate risks [39]. Conversely, if workers are prone to risk seeking, they often underestimate risks and are more likely to produce unsafe behaviors.

As for the last phase of responding and taking action, attitude and subjective norm control are main determiners that affect workers’ behavioral choices [38]. Attitude plays an important role in the behavior of the decision makers [37]. Risk-averse attitudes make workers more inclined to perform safe behavior. Subjective norms refer to the “Perceptions of significant others’ expectations to the behavior” [1]. Influential individuals or groups play an important role in determining whether or not a particular action is taken by a worker.

After a worker takes an action, the consequences of the action will also update the attitude and experience. Goles et al. [40] found that past behavior brought positive experience. In addition, the past behavior will lead individuals to have a more positive attitude toward such behaviors, which in turn will increase their willingness to implement the behavior again. Therefore, the effects of consequences of worker’s behavior on the experience and attitudes are considered in this paper.

#### 2.1.2. Social Group and Social Organizational Factors

A large body of literature indicates that social groups (managers, coworkers, and foremen) have an important influence on the unsafe behavior of construction workers. The influence of coworkers on workers is from two sides. On the one hand, coworkers could remind their peers not to perform an unsafe act so that workers behave safely [41]. On the other hand, coworkers’ violations of safety rules or standards may be imitated by their peers, which has a dramatically adverse impact on individual safety behavior [42]. Managers have influences on workers in various ways. Workers’ income is highly related to the manager’s evaluation of their work performance [22], so workers have to choose their behavior according to the manager’s safety attitude. Moreover, oral care and safety feedback of managers have a greater impact on workers’ safe behavior [24,43]. The foreman is normally accepted as a central role in a group and is authorized to take compulsory measures (e.g., prizes, supervision, and penalty) to control the crew [23]. In most cases, the foreman is considered as the first level supervisor [23], and his or her management functions are fully considered in the previous studies. However, most foremen tend to directly undertake construction tasks and are also special workers [44]. The double roles of foremen as managers and operators are not clearly distinguished by researchers.

Managers and foremen often manage and control workers’ unsafe behaviors through various social organizational factors [37,45,46]. Previous literature emphasized the important impact of social organizational factors such as safety training, communication, and behavior feedback on workers [13,47,48]. Most of these, however, use cross-sectional study designs. Cross-sectional study design may limit causal conclusions [49]. It is difficult to reveal the mechanism of the influence of social organizational factors on workers. A cognitive mechanism is an internal process of workers’ behaviors. The influence of social organizational factors should be analyzed from the perspective of cognitive mechanisms, and they affect workers’ unsafe behavior. Through literature research on accident investigation, safety culture, safety management, and social psychology, five main social organizational factors and their impact on workers’ cognitive process factors are summarized as follows.

Safety communication refers to the degree, frequency, and effectiveness of the information exchange on safety problems between workers and management [50]. Safety communication has an important impact on safety knowledge [50,51]. If workers communicate with managers, foremen, and coworkers adequately about safety issues, they will know the importance of safety and which behaviors are unsafe. This will enhance their safety knowledge. In addition, face-to-face communication can greatly improve workers’ safety awareness because repeating safety reminders and warnings and persuasion are crucial to workers’ safety awareness improvement, which in turn leads to a higher level of safety behavior [52].

Safety training refers to the frequency, effectiveness, and thoroughness of training provided to workers to avoid safety accidents [30]. Safety training can improve workers’ safety information and safety awareness [53,54]. By participating in the safety training of managers, workers can better grasp the actual information on the site and improve their safety awareness. In addition, training proved to be an important source of knowledge [55]. In safety meetings, managers read safety manuals and safety regulations to workers so that they can better know which behaviors are dangerous and their safety awareness is promoted.

Behavior feedback refers to the feedback of management on workers with financial reward, punishment, verbal praise, or criticism [30]. Workers understand the thoughts of managers and foremen through behavior feedback, which is beneficial for forming subjective norms for workers. The behavior feedback can also influence the workers’ safety attitude and help them to understand the degree of risk acceptance of the foreman and the manager. If a worker’s safe behavior results in positive feedback, such as material reward or recognition, the worker will think it is more worthwhile behaving safely, and in this case, the safe behavior is reinforced [15]. Conversely, if a worker receives less frequent positive feedback, he or she may not know whether his or her behavior is correct or not, and his or her safety performance may be poor. 

The demonstration role indicates that workers often regard the foreman and courageous workers as their role models. This means that workers will observe and imitate the behaviors of foremen and coworkers to form their own subjective norm. Choudhry and Fang [22] found that some construction workers worked unsafely just to show that they were “tough guys”. The “tough guy” has a demonstration role for other workers. Foremen were safety role models for their crew: if foremen overlook some safety issue, the chance of other workers to copy will increase. 

Social identity is defined as “part of an individual’s self-concept which derives from his knowledge of his membership of a social group together with the value and emotional significance of that membership” [56]. If workers maintain a strong social identity with managers and foremen, the behavior feedback and demonstration role of managers and foremen can better help workers form a subjective norm.

**Figure 1:**
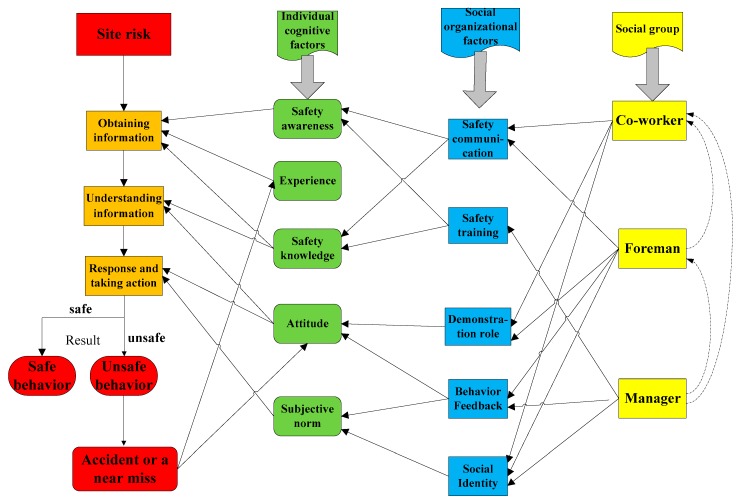
Framework of sociocognitive process of construction workers.

Therefore, five social organizational factors, including safety communication, safety training, demonstration role, behavior feedback, and social identity greatly affect worker’s individual cognitive factors. Combined with the workers’ cognitive factors, the complete sociocognitive model of construction workers’ unsafe behaviors is shown in Figure 1. 

In fact, in addition to directly affecting the unsafe behavior of the construction workers, managers also indirectly manage workers by affecting foremen and coworkers, and this impact is also implemented through safety communication and safety training. Similarly, the foreman will indirectly affect workers by affecting the coworkers. Such an impact is described in Figure 1 (the dashed line).

### 2.2. Agent-Based Modeling

Agent-based modeling, a computer simulation technique that allows the examination of how system rules and patterns emerge from the behaviors of individual agents. ABM is regarded as a modeling technique that matches more closely with the real-world situation than traditional equation-based modeling (EBM) [57], and it can explain the emergence of unsafe behavior of construction workers. Given that each project is a complex system involving different people, equipment, and activities, ABM has been considered an effective simulation technique [58]. ABM has been widely applied to process control, communication, transportation systems, medical, and other fields. In the construction industry, ABM has been most widely utilized to study production issues. Kim developed a multi-agent-based simulation system to evaluate the traffic flow of construction equipment in construction sites [59]. Ahn at al. [60] simulated the absence of construction workers and proposed one way to test whether the ABM model is effective. Goh et al. [61] proposed a hybrid simulation framework based on earthmoving to facilitate the inclusion of safety management considerations into the simulation of construction activities. In recent years, due to the complexity and dynamics of the construction site environment, traditional safety management methods are not so effective. Researchers have been trying to locate the causes of accidents through simulation. The ABM method has gradually been applied to the field of construction safety management. Sharpanskykh and Stroeve [62] proposed a new, formal, agent-based approach to study the relations between safety culture and formal and informal organizational structures and processes. Choi et al. [39] used an ABM method to study management norms, workgroup norms, and the relationship between security attitudes and unsafe behavior. Zhang et al. [26] established a model of scaffolding workers’ unsafe behaviors and explored the impact of management measures on workers’ safety performance. Ji et al. [63] adopted the ABM method to simulate the scene of reinforcing steel bars on the construction site, and proposed the support of the workers can reduce the occurrence of the nonfatal incidents. Liang et al. [42] developed a hybrid simulation approach that integrated System Dynamics (SD) and ABM to study the social contagion effect of safety violations within a construction crew. These studies have made great contributions to the field of construction safety simulation [49]. Previous ABM related studies often focused on the safety status of one job type on the construction site and did not represent the safety status of the entire construction site. Moreover, when studying workers’ unsafe behaviors, they are usually only considered from the social aspects of workers’ mutual assistance, management, standardization, and safety culture and ignoring the exploration of workers’ cognitive processes. Based on the previous study, this research supplements workers’ cognitive processes to better investigate the impact of social groups and social organizational factors on workers’ cognitive processes and unsafe behaviors.

## 3. Methodology

### 3.1. Framework

This research proposes an ABM simulation approach to understand construction worker’s sociocognitive processes. First, the workers’ sociocognitive process model is developed. In general, an agent-based model consists of three elements: (1) agent’s property, behavior, and environment; (2) agent’s relationships and interactions with the environment; and (3) agent’s relationships and interactions with other agents [33]. Therefore, the description of the model will be based on the above three components. Section 3.2 defines agents types and their relationships. Section 3.3 introduces workers’ individual cognitive process model, including the agent’s cognitive attributes, behaviors, and interactions with the environment (hazard and risk). Section 3.4 introduces the sociocognitive process of workers in interaction with managers, foremen, and workers. Secondly, the initialization of the ABM simulation is introduced in Section 3.5, including the simulation process and the determination of the initial value. The initial value is determined through survey interviews and empirical data. Third, a validation test is performed in Section 3.6 in order to ensure the quantitative and qualitative consistency of the model with the actual situation. Finally, simulation results were obtained using MATLAB simulation. Hardcoded Matlab program was the most widely used simulation tool [64]. The simulation result can be used for further analysis. 

### 3.2. Defining the Agent’s Types and Relationships

**Figure 2:**
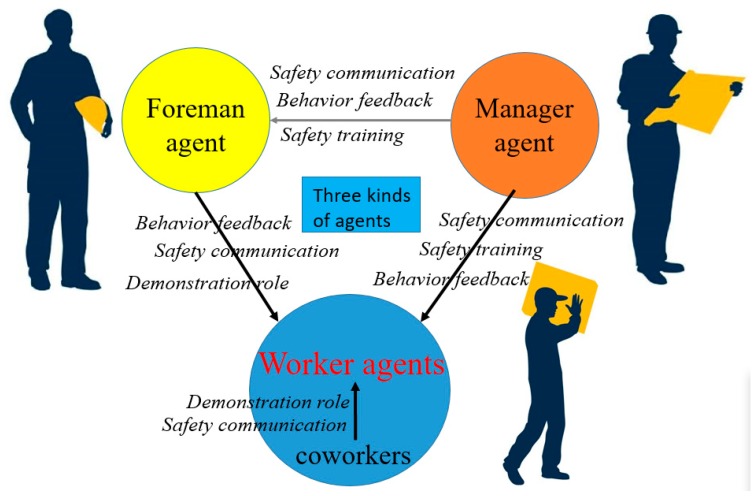
Relationship of worker, coworker, foreman, and manager.

In previous studies, the site agents have been divided into different types [26,39]. They mainly take the manager and the managed person as the dividing standard. Little research categorizes foremen into one category for discussion. As a special management group, the foreman whose safety performance is evaluated by the manager is responsible for workers’ unsafe behavior and often undertakes the construction task directly. The foreman has a dual status of manager and managed person. Taking into account the particular identity of foreman, agents in construction sites are divided into three categories: manager, worker, and foreman. There are two ways through which managers can influence workers. One is that managers interact directly with workers through management measures such as safety training, safety communication, and behavior feedback. The other is that managers indirectly influence workers through foremen. Managers will also provide safety training and safety communication to the foreman. As the connecting bridge between managers and workers, the foreman is responsible for supervising workers’ behavior; meanwhile, his/her behavior is also being supervised by managers [65]. Foreman’s behavior feedback, demonstration role, and safety communication have a significant impact on workers’ safety behavior. Coworkers and workers belong to the same agent type, and they have the same attributes as the worker agent. Within the agent of worker, the coworkers’ safety communication and behavior feedback have an important impact on workers’ unsafe behavior. The relationship among the three types of agents is shown in Figure 2.

### 3.3. Worker’s Individual Cognitive Model

**Figure 3:**
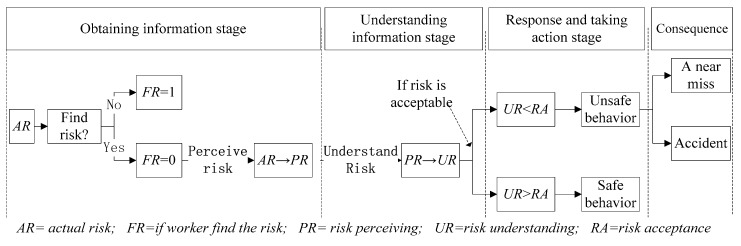
The worker’s individual cognitive process.

The worker’s individual cognitive model includes three stages: obtaining information, understanding information, and responding and taking action. When external risk information appears, workers firstly judge whether they can find the risk information, then perceive risk information, understand risk information, judge whether the risk is acceptable, and finally choose safe or unsafe behaviors. If workers perform unsafe behaviors, there will be two situations: near-miss accident or accident. The logic of the worker’s individual cognitive process is shown in Figure 3.

#### 3.3.1. Stage of Obtaining Information

In the obtaining information stage, with a low level of safety knowledge, inexperienced workers may not have the ability to detect a surrounding hazard and thus may not recognize the risk [30]. With a low level of safety awareness, workers may not expect hazards existing nearby; thus they may not search for hazards intentionally or not be able to detect hazards when searching unintentionally [17]. Therefore, if workers have both high safety knowledge and high safety awareness, they are more likely to find the danger; otherwise, they will probably not locate danger. The model is calculated using Equation (1).

(1) $$FR_{i}^{t}=\left\{ \begin{array}{l} 0\;Rand(0,1)\geq SA_{i}^{t}\times KL_{i}^{t}\\ 1\;Rand(0,1)<SA_{i}^{t}\times KL_{i}^{t} \end{array}\right.$$

$SA_{i}^{t}$ = worker *i*’s safety awareness at time *t*. The greater the safety awareness, the stronger the ability of workers to search for risks. $KL_{i}^{t}$ = worker *i*’s safety knowledge at time *t*. The greater the safety knowledge, the greater the workers’ ability to identify risks. Only when the random number generated is less than the product of $SA_{i}^{t}$ and $KL_{i}^{t}$, the worker can find the danger; otherwise, the danger cannot be found. $FR_{i}^{t}=0$ means the worker *i* does not find danger at time t, and $FR_{i}^{t}=1$ means the worker *i* find the danger at time *t*.

When the worker does not find the risk, $PR_{i}^{t}=0$ means the worker *i* does not perceive the risk at time *t*. When workers find the risk, workers’ perception of potential dangers come from the external environment and the information extraction of temporary memory and the knowledge base [66]. Therefore, the workers’ information perception model is calculated adopting Equation (2),

(2) $$PR_{i}^{t}=\left\{ \begin{array}{l} \lambda \frac{1}{t-1}\sum_{k=1}^{t-1}PR_{i}^{k}+(1-\lambda )\;AR_{i}^{t}\;(FR_{i}^{t}=1)\\ 0\;(FR_{i}^{t}=0) \end{array}\right.$$

where $PR_{i}^{t}$ = worker *i*’s risk perceiving at time *t*; $\frac{1}{t-1}\sum_{k=1}^{t-1}PR_{i}^{k}$ = worker *i*’s previous risk perception. Worker *i*’s previous risk perception will become a source of temporary memory and knowledge base for the next day. $AR_{i}^{t}$ = actual risk that worker *i* encounters at time *t*. λ indicates the worker *i*’s risk perception awareness. The stronger the risk perception experience, the greater the dependence of workers on past experience.

#### 3.3.2. Stage of Understanding Information

In the phase of the understanding information stage, the construction worker calculates the risk and the level of income brought by the behavior. According to bounded rationality theory, the limitation of an individual’s cognitive process may lead to construction workers’ underestimating or overestimating of the risk. The risk of worker perception and understanding may be different. Workers’ safety knowledge and attitudes will affect the understanding of risks [38,39]. The understanding coefficient is related to the safety attitude and safety knowledge. Thus, a worker’s risk understanding and risk understanding coefficient are calculated using Equation (3) and Equation (4).

(3) $$UR_{i}^{t}=p_{i}^{t}PR_{i}^{t}$$

(4) $$p_{i}^{t}=p_{i}^{t-1}-a1(AT_{i}^{t}-AT_{i}^{t-1})+a2(KL_{i}^{t}-KL_{i}^{t-1})$$

$UR_{i}^{t}$ = worker *i*’s risk understanding at time *t*. $p_{i}^{t}$ = worker *i*’s risk understanding coefficient at time *t*. $p_{i}^{t}>1$ means worker *i*’s risk understanding is higher than the risk perceiving at time *t*. $p_{i}^{t}<1$ means worker *i*’s risk understanding is lower than the risk perceiving at time *t*. $AT_{i}^{t}$ = worker *i*’s safety attitude at time *t*. Safety attitude refers to an individual’s tendency to take or avoid risk [39], with a range of 0 to 1 (risk adverse–risk seeking). $KL_{i}^{t}$ = worker *i*’s safety knowledge at time *t*. In this paper, when a worker’s safety attitude becomes risk seeking compared to the previous day, the risk understanding coefficient is correspondingly reduced, and the role of underestimation of risk is enhanced. Conversely, when a worker’s safety attitudes are more risk aversion than the day before, the risk understanding coefficient is correspondingly enhanced and the underestimation of risk is weakened. On the other hand, when workers have more safety knowledge, it means they will have a deeper understanding of the hazard and will overestimate the perceived risk. The impact of safety attitudes and safety knowledge on risk understanding may be different. a1 and a2 respectively indicate the degree of influence of changes in safety attitudes and safety knowledge on workers’ risk understanding.

#### 3.3.3. Stage of Responding and Taking Action 

During the responding and taking action stage, workers choose to perform safe or unsafe behavior. Cordell [67] points out that risk acceptance is related to safety attitudes. At the same time, subjective norms regulate workers’ risk acceptance; the risk acceptance is therefore calculated using Equation (5).

(5) $$RA_{i}^{t}=a3AT_{i}^{t}+a4SB_{i}^{t}$$

$RA_{i}^{t}$ = worker *i*’s risk acceptance at time *t*. $SB_{i}^{t}$ = worker *i*’s subjective norm at time *t*. a3 = proportional coefficient of worker’s attitude and risk acceptance. a4 = proportional coefficient of worker’s subjective norm and risk acceptance. Then, the model judges whether the worker performs unsafe behavior. In this paper, risk understanding being higher than risk acceptance means the worker considers the risk as unacceptable and adopts safe behavior. Otherwise, the worker considers the risk as acceptable and adopts unsafe behavior. The model is shown in Equation (6).

(6) $$UB_{i}^{t}=\left\{ \begin{array}{l} 0\;(\mathrm{if}\;RA_{i}^{t}<UR_{i}^{t})\\ 1\;(\mathrm{if}\;RA_{i}^{t}>UR_{i}^{t}) \end{array}\right.$$

$UB_{i}^{t}$ is a binary indicator of whether worker *i* has taken unsafe actions at time *t*. ${UB}_{i}^{t}=1$ means worker *i* performs unsafe behavior at time *t*. $UB_{i}^{t}=0$ means worker *i* performs safe behavior at time *t*. 

Heinrich [9] pointed out that the unsafe state of things (working environment) and the unsafe behavior of people and their interactions are the direct causes of accidents. When workers are in an unsafe work environment and they perform unsafe behavior at the same time, an accident is considered to occur. Otherwise, no accidents occur. The model is shown in Equation (7).

(7) $$accident_{i}^{t}=\left\{ \begin{array}{l} 0\;(\mathrm{if}\;(UB_{i}^{t}=1,Rand(0,1)>ER^{t})\;or\;(UB_{i}^{t}=0))\\ 1\;(\mathrm{if}\;UB_{i}^{t}=1,Rand(0,1)<ER^{t}) \end{array}\right.$$

$ER^{t}$ = unsafe state of the working environment. The larger the value, the greater the probability that the environment is in an unsafe state; the smaller the value, the smaller the probability that the environment is in an unsafe state. $accident_{i}^{t}$ means whether the accident happened. $accident_{i}^{t}=1$ means an accident occurred. $accident_{i}^{t}=0$ means no accidents occurred.

The consequences of the accident will update workers’ safety attitudes [40]. If workers perform unsafe behaviors but no danger occurs, they become inclined to risk seeking; if workers perform unsafe behavior and an accident happened, they become inclined to risk aversion; if the workers do not perform unsafe behavior, then the risk attitude remains unchanged. The model is shown in Equation (8).

(8) $$AT_{i}^{t+1}=\left\{ \begin{array}{l} AT_{i}^{t}\;(\mathrm{if}\;UB_{i}^{t}=0)\\ AT_{i}^{t}-c1\;(\mathrm{if}\;UB_{i}^{t}=1,accident_{i}^{t}=1)\\ AT_{i}^{t}+c2\;(\mathrm{if}\;UB_{i}^{t}=1,accident_{i}^{t}=0) \end{array}\right.$$

where c1 represents the decrease in attitude and c2 represents the increase in attitude.

### 3.4. Model of Coworker, Foreman, and Manager Interaction 

#### 3.4.1. Interaction Model in the Stages of Obtaining Information and Understanding Information 

Worker’s safety awareness is affected by coworkers, foremen, and managers. If coworkers and foremen often communicate with workers, the workers’ understanding of actual risks will be improved [52]. At the same time, if adequate safety training has been provided to the practitioner by the accountable manager during daily operation, the worker’s safety awareness will be also greatly improved [53]. The model is shown in Equation (9).

(9) $$SA_{i}^{t}=\sum_{j=1}^{3}CN_{ij}^{t}CI_{j}+SA0$$

SA0 = initial safety awareness, indicating the safety awareness of workers before they enter the project. $CN_{ij}^{t}$ indicates the frequency at which workers communicate with workers and foremen or participate in manager training at time *t*. *j* = 1 means the frequency of communication with workers. *j* = 2 means the frequency of communication with the foreman. *j* = 3 means the frequency of participation in manager training. $CI_{j}$ represents the promotion of workers’ safety awareness by communicating with workers and foremen or participating in manager training. In this article, safety meetings are held before workers start work, and safety communications take place while workers are at work or after taking action.

In the stage of understanding information, workers’ safety knowledge and attitudes affect workers’ understanding of risks [38,39]. If coworkers and foremen often communicate with workers, the workers’ safety knowledge will be improved. At the same time, if adequate safety training has been provided to the practitioner by the accountable manager during daily operation, the worker’s safety knowledge will be also greatly improved. The model is shown in Equation (10).

(10) $$KL_{i}^{t}=\sum_{j=1}^{3}CN_{ij}^{t}CI_{j}^{\prime }+KL0$$

KL0 = initial safety knowledge, indicating the safety knowledge of workers before they enter the project. $CI_{j}^{\prime }$ represents the promotion of workers’ safety knowledge by communication with workers and foreman or participating in manager training.

#### 3.4.2. Interaction Model in the Stage of Responding and Taking Action 

In the responding and taking action stage, the risk acceptance of workers is not only affected by the subjective attitude of workers but also by workers, foremen, and managers. Choi et al. [56] pointed out that workers’ risk acceptance is affected by management norm, workgroup norm, and risk attitude. In a workgroup, not only coworkers, but also the foreman, has a great influence on the risk acceptance of workers. Manager norm, foreman norm, and coworker norm will all form subjective norm for workers, which in turn affects workers’ risk acceptance. Therefore, the worker’s risk acceptance model is shown in Equation (11).

(11) $$RA_{i}^{t}=(1-w)AT_{i}^{t}+w\left(aWN_{i}^{t}+bFN_{i}^{t}+cMN_{i}^{t}\right)+\varepsilon$$

w = social identity, indicating the extent to which workers identify with others. $WN_{i}^{t}$ = worker *i*’s coworker norm at time *t*. $FN_{i}^{t}$ = worker *i*’s foreman norm at time *t*. $MN_{i}^{t}$ = worker *i*’s manager norm at time *t*. *a*, *b*, and *c* indicate the weight coefficient of the coworker norm, the foreman norm, and the manager norm, respectively. a+b+c=1. ε = random impact factor, represents the random fluctuation of the risk acceptance due to unexplained outside influence.

**Figure 4:**
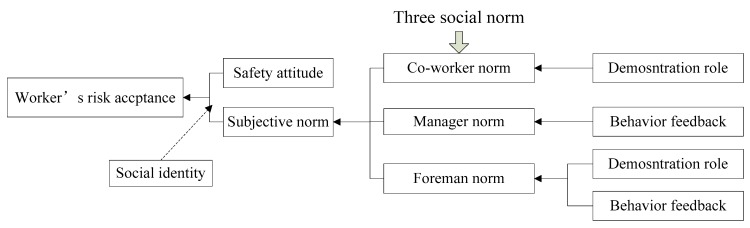
The schematic diagram of foreman norm, coworker norm, and manager norm.

In fact, coworker norm is mainly determined by coworkers’ demonstration role, and manager norm is largely determined by manager’s behavior feedback [39]. In this article, the foreman norm has been added. The foreman has dual effects on workers through behavior feedback and demonstration role. The schematic diagram is shown in Figure 4.

##### Foreman Norm

In the model, workers perceive the foreman norm by perceiving the foreman’s risk acceptance. Stored information is used to perceive social norms [60]. Therefore, the current foreman norm is determined by the previous day’s foreman norm and the perceived risk acceptance of the foreman at this stage, as shown in Equation (12). In the model, workers perceive the risk acceptance of the foreman in two ways, i.e., through the foreman’s demonstration role and through the foreman’s behavior feedback. The demonstration role means the workers follow the actions of foreman. As is shown in Equation (13), if the worker observes that the foreman performs unsafe behavior, then he may perceive that the foreman’s risk acceptance is higher than the actual risk, and therefore his risk acceptance will increase. Conversely, if the worker observes that the foreman performs safe behavior, then he may perceive that the foreman’s risk acceptance is lower than the actual risk, and thus his risk acceptance will decrease. The behavior feedback indicates that the foreman may give either positive or negative feedback based on workers’ actual performance. If worker *i* performs safe behavior at time *t* and receives positive feedback, then he may think that the foreman is encouraging him to perform safe behavior, and accordingly, his risk acceptance level will decline. If the worker performs safe behavior without receiving positive feedback, his risk acceptance level remains unchanged. If the worker conducts dangerous operations and receives negative feedback, he may believe that his risk acceptance level is too high and his unsafe behavior is unacceptable for the foreman, and as a result, his risk acceptance level will drop off. If the worker performs unsafe behavior without receiving negative feedback, he may think that his risk acceptance is appropriate and his unsafe behavior is acceptable for the foreman, and accordingly, his risk acceptance will improve. The feedback effect is calculated using Equation (14)

(12) $$FN_{i}^{t}=(1-m)FN_{i}^{t-1}+m(dPFRA_{i}^{t}+(1-d)PFA_{i}^{t})$$

$PFRA^{t}$ = the demonstration role of the foreman to workers at time *t*. $PFA_{i}^{t}$ = the foreman’s behavior feedback on worker *i*’s behavior at time *t*, and *d* = the proportion of foreman’s demonstration role. *m* = worker’s memory level, indicating the impact on the memory of the previous stage.

(13) $$PFRA=\left\{ \begin{array}{l} Rand(AR_{i}^{t},1)\;(\mathrm{if}\;UB\prime_{k}^{t}=1)\\ Rand(0,AR_{i}^{t})\;(\mathrm{if}\;UB\prime_{k}^{t}=0) \end{array}\right.$$

(14) $$PFA_{i}^{t-1}=\left\{ \begin{array}{l} FN_{i}^{\left(t-1\right)}\;(\mathrm{if}\;UB_{i}^{\left(t-1\right)}=0,PFF_{i}^{\left(t-1\right)}=0)\\ Rand(0,UR_{i}^{\left(t-1\right)})(ifUB_{i}^{\left(t-1\right)}=0,PFF_{i}^{\left(t-1\right)}=1)\\ Rand(0,UR_{i}^{\left(t-1\right)})(ifUB_{i}^{\left(t-1\right)}=1,FF_{i}^{\left(t-1\right)}=1)\\ Rand(UR_{i}^{\left(t-1\right)},1)(ifUB_{i}^{\left(t-1\right)}=1,FF_{i}^{\left(t-1\right)}=0) \end{array}\right.$$

$UB\prime_{k}^{t}=1$ means foreman *k* performs unsafe behavior at time *t*. $UB\prime_{k}^{t}=0$ means foreman *k* performs safe behavior at time *t*. $PFF_{i}^{\left(t-1\right)}$ means whether the foreman gives positive feedback to the worker’s safe behavior at time *t*-1 (1 = gives feedback and 0 = no feedback). $FF_{i}^{\left(t-1\right)}$ means whether the foreman gives negative feedback on worker *i*’s unsafe behavior at time *t*-1 (1 = gives feedback and 0 = no feedback).

##### Coworker Norm 

In the model, workers perceive coworker norms by perceiving the coworker’s risk acceptance. The current coworker norm is determined by the previous day’s coworker norm and the perceived risk acceptance of the coworker at this stage. The model is shown in Equation (15). Each worker perceives the coworker norm by observing the behavior of other workers. If worker *i* observes the coworker *k* performing unsafe behavior, he/she may believe that coworker *k* performs unsafe behavior because *k*’s risk acceptance is higher than actual risk, and accordingly, his risk acceptance will increase. If worker *i* observes coworker *k* performing safe behavior, he may consider that *k*’s risk acceptance is lower than the actual risk, and correspondingly, his own risk acceptance will decrease. The model is shown in Equation (16).

(15) $$WN_{i}^{t}=mWN_{i}^{t-1}+(1-m)(\frac{1}{k_{i}^{t}}\sum_{k=1}^{k_{i}^{t}}PRA_{ik}^{t})$$

(16) $$PRA_{ik}^{t}=\left\{ \begin{array}{l} Rand(AR_{k}^{t},1)\;(\mathrm{if}\;UB_{k}^{t}=1)\\ Rand(0,AR_{k}^{t})\;(\mathrm{if}\;UB_{k}^{t}=0) \end{array}\right.$$

$PRA_{ik}^{t}$ = the demonstration role of coworker *k* to worker *i* at time *t*. $UB_{k}^{t}$ means whether worker *k* performs unsafe behavior at time *t*. 0 = worker performs safe behavior. 1 = worker performs unsafe behavior. $k_{i}^{t}$ = the number of coworkers that worker *i* observed at time *t*. Since the foreman also bears the same labor work as the workers at the construction site, the cognitive process of the workers’ unsafe behavior also applies to the foreman.

##### Manager Norm

In the model, workers perceive manager norm by perceiving the manager’s risk acceptance. As is shown in Equation (17), the current manager norm is determined by the previous day’s manager norm and worker *i*’s perception of managers’ risk acceptance at this stage. Managers conduct safety-oriented interactive feedback that can effectively reduce workers’ unsafe behavior [68]. Workers perceive the manager’s risk acceptance through the manager’s behavior feedback. As is shown in Equation (18), the manager’s behavior feedback is similar to that of the foreman.

(17) $$MN_{i}^{t}=(1-\frac{1}{m})MN_{i}^{t-1}+\frac{1}{m}(PMA_{i}^{t})$$

(18) $$PMA_{i}^{t}=\left\{ \begin{array}{l} MN_{i}^{\left(t-1\right)}\;(\mathrm{if}\;UB_{i}^{\left(t-1\right)}=0,\;PMF_{i}^{\left(t-1\right)}=0)\\ Rand(0,UR_{i}^{\left(t-1\right)})(ifUB_{i}^{\left(t-1\right)}=0,PMF_{i}^{\left(t-1\right)}=1)\\ Rand(0,UR_{i}^{\left(t-1\right)})(ifUB_{i}^{\left(t-1\right)}=1,MF_{i}^{\left(t-1\right)}=1)\\ Rand(UR_{i}^{\left(t-1\right)},1)(ifUB_{i}^{\left(t-1\right)}=1,MF_{i}^{\left(t-1\right)}=0) \end{array}\right.$$

$PMA_{i}^{t}$ = the manager’s behavior feedback on worker *i*’s behavior at time *t*, and $PMF_{i}^{\left(t-1\right)}$ represents whether the manager gives positive feedback on worker *i*’s unsafe behavior at time *t*-1 (1 = feedback, 0 = no feedback). $MF_{i}^{\left(t-1\right)}$ represents whether the manager gives negative feedback on worker *i*’s unsafe behavior at time *t*-1 (1 = feedback, 0 = no feedback). After receiving feedback from the actions of the foreman or manager, workers are more aware of the dangers of risk, and their attitudes also tend to be safe.

### 3.5. Model Initialization

#### 3.5.1. Simulation Process

**Figure 5:**
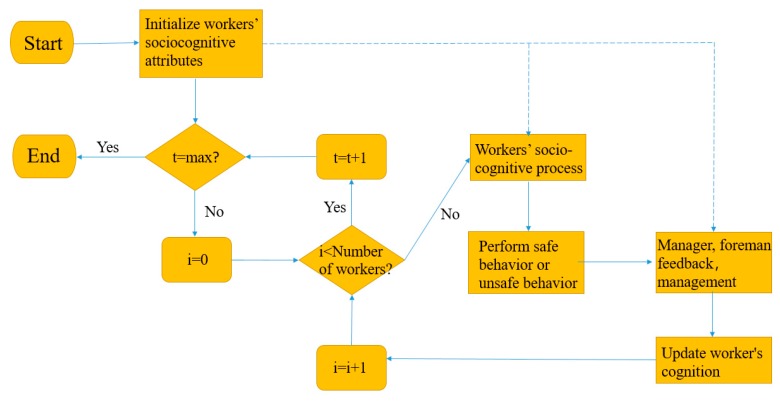
Simulation process.

The model continues with a day time step and means workers who perform safe or unsafe behavior based on the interaction between their cognitive process and the other groups (coworkers, foremen, and managers). Firstly, the model initializes the site conditions and workers’ attributes, begins to progress forward by a day, and simulates workers’ safety behavior. During each time step, every worker has a chance to perform safe behavior or unsafe behavior. When all the workers are simulated, the model moves on to the next day. Figure 5 shows the simulation process.

#### 3.5.2. Initializing Parameters

There are 100 workers on the simulated construction site, consisting of five work groups with 20 workers in each work group. According to the study of Lu et al. [69], a foreman usually supervises 20 workers in one group. The duration of the simulation is set at 280 days, based on an actual engineering project. The range of worker’s safety attitudes is assigned based on the uniform distribution from 0.4 to 0.9. The uniform distribution tends to be the most appropriate when the distribution is unknown [70]. The mean value of the worker’s safety attitude is determined to be higher than 0.5, reflecting that workers’ safety attitude tends to be risk seeking. The range of worker’s initial risk understanding coefficient is assigned based on the uniform distribution from 0.6 to 1.2. The mean value of the worker’s initial risk understanding coefficient is less than 1.0 in order to reflect construction workers’ tendency to underestimate the perceived risk and overestimate their ability to control the environment [37]. The minimum value of the risk understanding coefficient is determined according to the result of Choi et al. [39] and Shin et al. [71]. 

The initial safety knowledge and safety awareness are set as 0.8, which means that workers have the knowledge to deal with risk and the awareness to discover risks in 80% of the cases. The frequency of safety meetings is set at 0.5, indicating that safety training meetings will be held every two days. The frequency of negative feedback is set as 0.6, meaning that the probability for workers receiving negative feedback from managers every day is 60%. The positive feedback frequency is set as 0.1 in order to reflect that there is less positive feedback from managers and foremen. In the baseline model, the weight of foreman norm, manager norm, and coworker norm are 0.45, 0.35, and 0.2, respectively, which means that workers are more willing to listen to the foremen on the site, followed by managers, and then coworkers. Then, the value of social identity is set as 0.5 in the baseline model, meaning that workers have a 50% chance of consulting others and a 50% chance of consulting themselves. Lastly, the distribution of the actual risk level for the baseline model is set at a modest level by a triangular distribution of (0.1, 0.5, 0.9). The lowest value is 0.1 and the highest value is 0.9 to avoid extreme low and high conditions [72]. Triangular distributions are used to simulate risk in previous research [72]. The initial values of the model are shown in Table 1. In the baseline model, all variables are simulated according to the initial values to reflect workers’ sociocognitive processes. When examining the impact of different social organizational factors on workers’ unsafe behavior, the default values of the social organizational factors are set according to specific scenarios to represent different management strategies. Note that the initial values of the model are exemplary, and the initial values of the model can be changed to simulate different engineering projects.

### 3.6. Model Validation

The validation process is to determine whether the model provides a reasonable and realistic representation of the real world to solve the research problem. A clear description of the phenomena to be explained by the model and the testing of the simplest and realistic proxy behavior rules are the key to successful ABM validation [73]. In this regard, Zeigler et al. [74] proposed three different types of validity: replicative validity (i.e., the model “matches data already acquired from the real system”), structural validity (i.e., the model “truly reflects the way in which the real system operates”) and predictive validity (i.e., the model “matches data before data acquired from the real system”). This paper mainly studies the influence of social groups and social factors on unsafe behaviors, rather than specifically predicting the occurrence of unsafe behaviors. Therefore, only replication validity and structural validity are tested. For the replication validity, Axtell and Epstein [75] summarized the four different levels of performance of an agent-based model. The lowest level of model performance (i.e., Level 0) is present when “the agent behavior rule is in qualitative agreement with the micro behavior.” Level 1 is achieved when “the model behavior is in qualitative agreement with empirical macro-structures.” Level 2 is present when “the model behavior is in quantitative agreement with empirical macro-structures.” The highest level of the model performance (i.e., Level 3) is achieved when “the model behavior is in quantitative agreement with empirical micro-structures.” Level 3 (i.e., quantitative correspondence of an agent’s behavior in the simulation with the micro level, actual human behavior) would not be realistic in the human behavior simulation due to the inherent uncertainty in human behavior and the random events in reality [76]. Therefore, qualitative and quantitative agreement is used in this paper to facilitate the model satisfying the criteria of Level 2 models. Firstly, the model results were compared with previous empirical studies to test the qualitative consistency of the model. Then, unsafe behavior ratio, nonfatal injuries data, and accident data were used to verify the quantitative consistency of the model.

**Figure 6:**
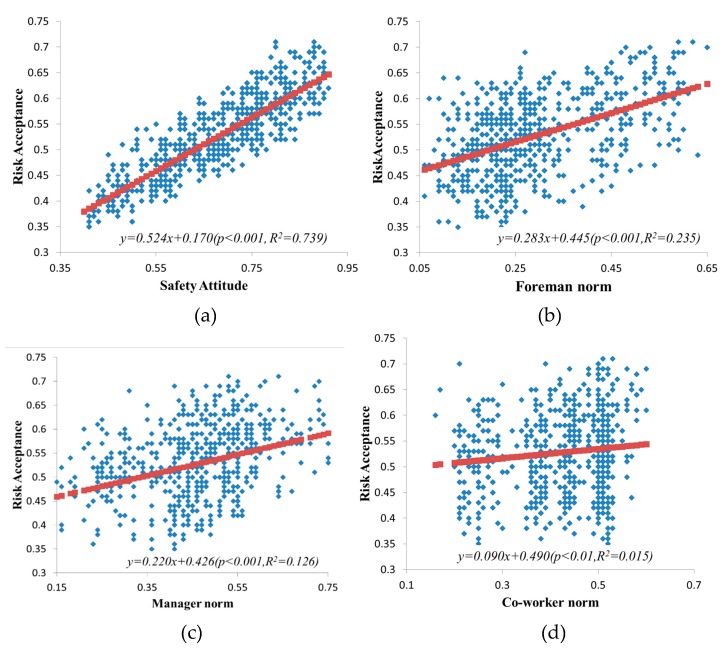
Relationships among attitude, coworker norm, foreman norm, manager norm, and risk acceptance in the baseline model.

The qualitative agreement of the replicative validity was first examined. The test result also reiterated the role of personal attitude in workers’ risk acceptance level. As shown in Figure 6a, the relationship between risk acceptance and safety attitude can be expressed by the formula: y = 0.524x + 0.170. A coefficient of 0.524 means that safety attitude has a positive relationship with workers’ risk acceptance (R^2^ = 0.739; *p* < 0.001). As workers show risk-seeking attitudes (i.e., closer to 1), they are more tolerant of risk and have a higher level of risk acceptance. The study also revealed the impact of social factors on workers’ risk acceptance. As is shown in Figure 6b,c, foremen norm and manager norm have a positive correlation with workers’ risk acceptance (R^2^ = 0.235; *p* < 0.001) (R^2^ = 0.126; *p* < 0.001). Through the feedback and management of workers’ unsafe behavior by the foreman and managers, the workers observe the lower risk acceptance, which will reduce their own risk acceptance accordingly. As Figure 6d illustrates, coworker norm has a slight positive relation with worker’s risk acceptance level (R^2^ = 0.015; *p* < 0.01). The possible reason is that the impact of the coworker norm on the workers is not all positive. When the coworkers perform unsafe behaviors, the workers are likely to imitate, which has a negative impact on workers. For managers and foremen, the impact of their management norms on workers are often positive, and managers and foremen have fewer negative effects on workers’ unsafe behavior.

It is noted that the values of R^2^ obtained from the above three linear regressions are not very high. This means that there may be more factors affecting workers’ risk acceptance, and more variables should be included in the regressions. Nevertheless, the significant *p*-value in this regression still indicates a positive relationship between these three variables (i.e., manager norm, foreman norm, coworker norm) and worker’s risk acceptance. 

Pearson correlation is used to test the influence of social organization factors on workers’ individual cognitive factors. Statistically, Pearson’s correlation coefficient is one of the methods used for a broad class of relationships among variables [77], evaluating the strength of the relationship between the two vectors, based on the covariance matrix [78]. As shown in Table 2, the Pearson correlation coefficient of safety awareness and safety communication is 0.705, within the scope of 0.6–0.8, and could be therefore considered as a significant positive correlation. When workers communicate with the foreman, they are more aware of the risks of unsafe behavior and can improve their safety awareness. Worker’s safety awareness has a positive relationship with the manager-organized safety training (Pearson correlation coefficient = 0.735). After participating in safety training, workers’ safety awareness has been greatly improved. The simulation results are consistent with the actual situation and previous research [52,53]. In addition, the simulation results reiterated the positive impact of safety training and safety communication on workers’ safety knowledge, which is qualitatively consistent with the macro level [51,55].

The simulation results are also compared with empirical data from previous studies to ensure quantitative agreement with empirical macrostructures (i.e., Level 2). The baseline model runs 100 times, and the average values of several significant indicators are listed in Table 3. Firstly, the average ratio of unsafe behavior is 0.326, which is very close to the findings of Sa et al. [79] and Fang and Wu [11], which is that about one-third of workers take unsafe behavior on the construction site. Secondly, the average rate of accidents (i.e., the number of accidents per 100 full-time workers) is 3.35, which is consistent with the national incidence rate of occupational injuries and illnesses in construction reported by the U.S. Bureau of Labor Statistics in 2016 (equal to 3.2) [80]. Finally, according to the research of Heinrich et al. [9], the ratio between near misses and accidents (major and minor accidents) is 300:30 [39], and the simulation result (9.47:1) is in line with the empirical data.

## 4. Result

### 4.1. Manager, Foreman, and Coworker Interaction Influence Baseline Model

**Figure 7:**
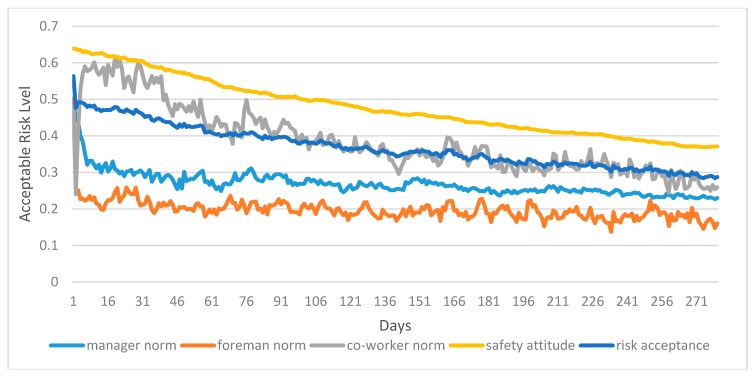
Changes in manager norm, foreman norm, coworker norm, safety attitude, and risk acceptance in the baseline model.

In total, 100 simulation runs were to explore the effects of the interaction between workers’ sociocognitive process and the coworkers, foremen, and managers on risk acceptance and unsafe behaviors across a simulated site. Figure 7 shows the changes in the mean of coworker norm, manager norm, foreman norm, worker’s safety attitude, and worker’s risk acceptance over time in the baseline model. The horizontal axis refers to time steps in the simulation, while the vertical axis represents the acceptable risk level. Coworker norm, manager norm, and foreman norm reflect the risk acceptance level of coworker, manager, and foreman. Safety attitude shows the risk acceptance level of workers’ own safety attitude. Risk acceptance reflects the risk acceptance level of workers under three social norms and the worker’s own attitude. As shown in Figure 7, worker’s risk acceptance level is lower than the risk acceptance of own safety attitude in the sociocognitive model, indicating the worker’s risk acceptance level is not only determined by his own safety attitude, but also by the coworkers, foreman, and the managers. By comparing the three types of social norms, the risk acceptance level of foreman norm and manager norm is lower than that of coworker norm. This fully proves that the manager norm and foreman norm are stricter than coworker norm. Nevertheless, workers’ risk tolerance is often higher than coworkers’ risk acceptance, which means that the impact of workers’ norms may not be positive.

**Figure 8:**
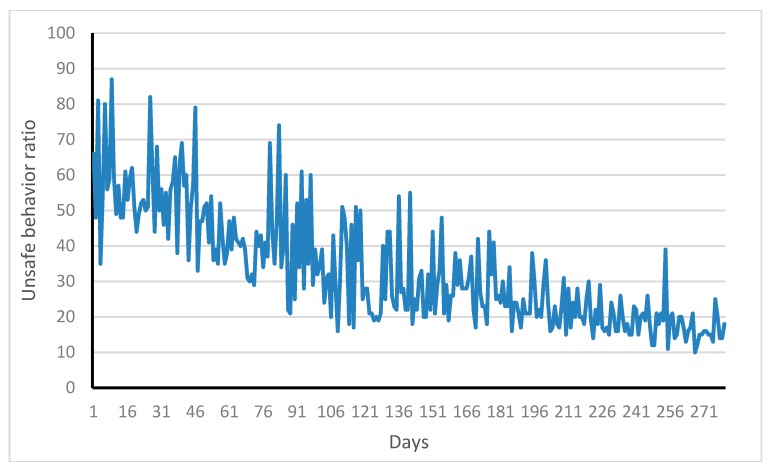
Changes in unsafe behavior of construction workers with time. (Note: unsafe behavior ratio = the number of unsafe behaviors/(the number of unsafe behaviors + the number of safe behaviors).

**Figure 9:**
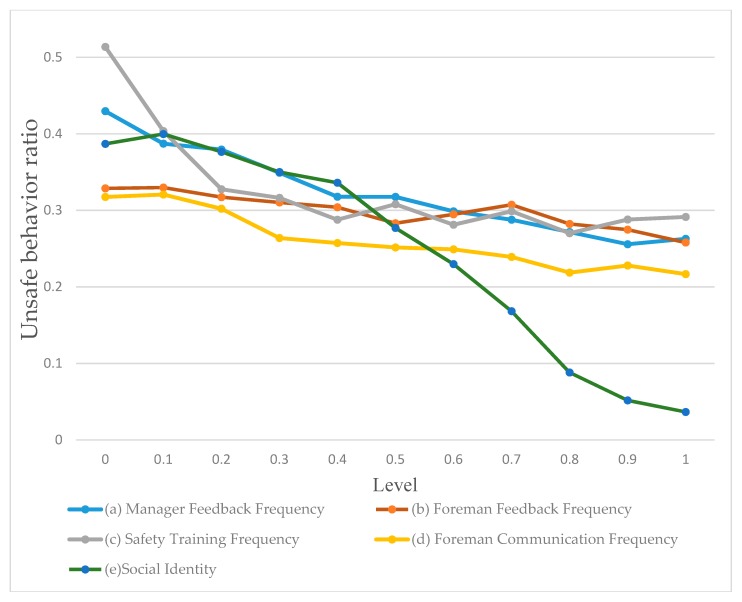
Changes in construction workers’ unsafe behavior under different management parameters.

Figure 8 shows the change of unsafe behavior ratio of workers under the interaction of managers, foremen, and coworkers. In this paper, as illustrated in Figure 8, workers’ unsafe behavior ratio decreases from 60% on day 0 to 20% on day 280. Workers’ unsafe behaviors are obviously under control after interacting with coworkers, foreman, and managers. A comparison of Figure 8 and Figure 9 shows that under social interaction, workers’ risk acceptance and unsafe behavior have decreased, indicating that social groups play an important role in workers’ safe behavior.

### 4.2. Not Considering the Single Social Norm or Foreman’s Demonstration Role 

In order to better understand the influence of foremen, managers, and coworkers on workers’ unsafe behaviors, this research excludes the effects of manager norm, and coworker norm, foreman norm, respectively, at each time. For example, if the influence of the foreman norm is not included, workers’ risk acceptance is determined by worker’s attitude, coworker norm, and manager norm, and foreman norm is not related to risk acceptance. The following formula will change from Equation (11) into Equation (19).

(19) $$RA_{i}^{t}=(1-w)AT_{i}^{t}+w\left(aWN_{i}^{t}+cMN_{i}^{t}\right)+\varepsilon \;(a+c=1)$$

Excluding manager norm and coworker norm is similar to excluding foreman norm. If one factor is excluded and there is a large change in workers’ unsafe behavior, it means that the factor has a major influence on workers’ unsafe behavior. 

Foreman norm is determined by demonstration role and behavior feedback. Previous literature rarely takes foreman’s demonstration role into consideration, but the effect of demonstration role is taken additional consideration in this paper. If the foreman’s demonstration role is excluded, the foreman norm is only determined by behavior feedback. In this situation, Equation (12) is changed into Equation (20) as follows.

(20) $$FN_{i}^{t}=(1-m)FN_{i}^{t-1}+mPFA_{i}^{t}$$

The simulation model runs 100 times for each variation. The change of unsafe behavior rate is shown in Table 4. When manager norm is excluded, workers’ unsafe behavior ratio and accident rate are much higher than the baseline model, emphasizing the importance of manager norm. However, when workers’ coworker norm is excluded, the unsafe behavior ratio and accident rate are similar to the baseline model, which means the effect of coworker norm on workers’ unsafe behavior is not significant. This is in line with the previous results [25]. When foreman norm is excluded, workers’ unsafe behavior ratio is 13.50% higher than the unsafe behavior ratio in the baseline model. The results underscore the importance of foreman norm. Under the role of foreman norm, workers’ risk acceptance becomes lower and they perform more safe behavior. When the foreman’s demonstration role is not taken into account, workers’ unsafe behavior ratio increases by 6.13% and accident rate increases by 1.79% over the baseline model. The results also highlight the impact of foreman’s demonstration role on workers’ unsafe behavior. The foreman’s demonstration role reduces the risk acceptance of workers, resulting in fewer unsafe behaviors. Comparing foreman norm with manager norm and coworker norm, it can be found that excluding the foreman’s norm, there is a higher proportion of unsafe behaviors and higher accident rates.

### 4.3. Causes of Cognitive Failure of Construction Workers

The cognitive process of construction workers is a dynamic process and may be affected by many factors. In order to explore causes of cognitive failure of construction workers, the main factors of construction workers’ sociocognitive process are analyzed. As shown in Table 5, Case 1, Case 2, and Case 3 describe three types of reasons for the failure of construction workers’ cognitive processes. Case 1 shows that the worker did not locate the risk. While Case 2 reveals that the worker underestimated the risk. In Case 3, however, the worker correctly estimated the risk but did not choose the safe behavior. On the other hand, Case 4 describes the failure of one stage of the worker’s sociocognitive process to be corrected under the social norm.

In Case 1, the worker’s *PR* is 0, which indicates the worker did not find the risk. An error occurred in the first stage of the worker’s cognitive process. *KL* and *SA* (*KL* = 0.832; *SA* = 0.832) are close to the initial value of *KL* and *SA* (initial value = 0.8), which means worker’s safety awareness and safety knowledge are insufficient. This is the direct reason for workers not finding risk. In addition, *ST* = 0 and *FCN = 0* mean the worker not participating in safety training and lacking communication with the foreman. This may be the reason that worker’s *KL* and *SA* are not enough.

In Case 2, the worker found the risk at the first stage of the cognitive process (*PR* = 0.522 > 0) but underestimated the risk (*UR* = 0.343 < *PR* = 0.522) in the second cognitive stage. Hence, an error occurred in the second stage of the worker’s cognitive process. The worker thought the risk was acceptable (*RA* = 0.475 > *UR* = 0.343) and took unsafe behavior (*UB* = 1). In addition, workers not receiving safety training (*ST* = 0) results in a lower safety knowledge (*KL* = 0.841), which may be the reason why workers underestimate the risk.

In Case 3, the worker found the risk at the first stage of the cognitive process (*PR* = 0.453 > 0), and he could correctly understand the risk in the second stage and overestimate the harm caused by the risk (*UR* = 0.573 > *PR = 0.453*). However, due to the high degree of worker’s risk acceptance (*RA* = 0.629), worker’s risk acceptance exceeds the risk understanding (*UR* = 0.573). The worker believes that the risks are acceptable and performs unsafe behavior (*UB* = 1) in the stage of responding and taking action. Thus, an error occurred in the third stage of the worker’s cognitive process. In addition, the manager norm (*MN* = 0.486) is highest compared to that in Case 2 and Case 3. This may be the reason for worker’s higher risk acceptance.

In Case 4, the worker found the risk at the first stage of the cognitive process (*PR* = 0.595 > 0) and underestimated the risk in the second stage (*UR* = 0.478 < *PR* = 0.595). Cognitive biases occurred. In the third stage of workers’ cognitive process, coworker norm, foreman norm, and manager norm all show low risk acceptance (*WN* = 0.305, *FN* = 0.237, *MN* = 0.150). By interacting with social groups, the worker’s risk acceptance was lower than worker’s risk understanding (*RA* = 0.403 < *UR* = 0.478), and the worker believes the risks are unacceptable and performs safe behavior (*UB* = 0) As a result, the worker’s cognitive biases have been corrected.

### 4.4. Analysis of Single Social Organizational Factors Influence

Zohar divides management actions into three categories: reactive action (RA), preventive action (PA), and prioritization (P) [81]. Reactive action is the action taken by a manager or foreman after a worker’s unsafe behavior. In this research, reactive actions include manager feedback, foreman feedback, and foreman communication. The frequency is used as a representation of the intensity of communication and feedback. The higher the feedback frequency, the greater the effect of the feedback. Preventive actions include manager safety training, which is carried out before workers start working. Prioritization refers to the social identity of workers, indicating the influence of social groups on workers. These five parameters represent possible managerial strategies to reduce workers’ unsafe behaviors at a construction site. When one parameter changes, other parameters remain as the value of the baseline model. Each parameter has 11 levels from 0 to 1 in the simulation: e.g., if the level of the manager’s feedback frequency is 0.1, it means that the probability that the worker receives the manager’s feedback on the day is 0.1. The effect of change of the five parameters on workers’ unsafe behavior ratio is shown in Figure 9.

Comparing these five social organizational factors, we see that social identity has the most significant effect on workers’ unsafe behaviors. With the increase of social identity, the proportion of workers’ unsafe behavior shows an obvious downward trend. Workers with higher social identity accept more management measures of social groups and are more likely to perform safe behaviors. The second most important factor is preventive action (manager safety training). When safety training frequency increases from 0 to 0.4, workers’ unsafe behavior greatly reduces. A little safety training can have a big impact on workers. When safety training frequency increases from 0.4 to 1, it shows a marginal effect that the proportion of unsafe behavior remains almost unchanged. This indicates that excessive safety training cannot effectively reduce workers’ unsafe behavior. As for reactive actions, manager feedback and foreman communication also play important roles in reducing workers’ unsafe behavior. They plot parallel lines on the figure, which means they have a similar impact on workers’ unsafe behavior. Among all five social organizational factors, foreman feedback frequency has the least influence on workers’ behavior.

### 4.5. Analysis of Paired Social Organizational Influence 

**Figure 10:**
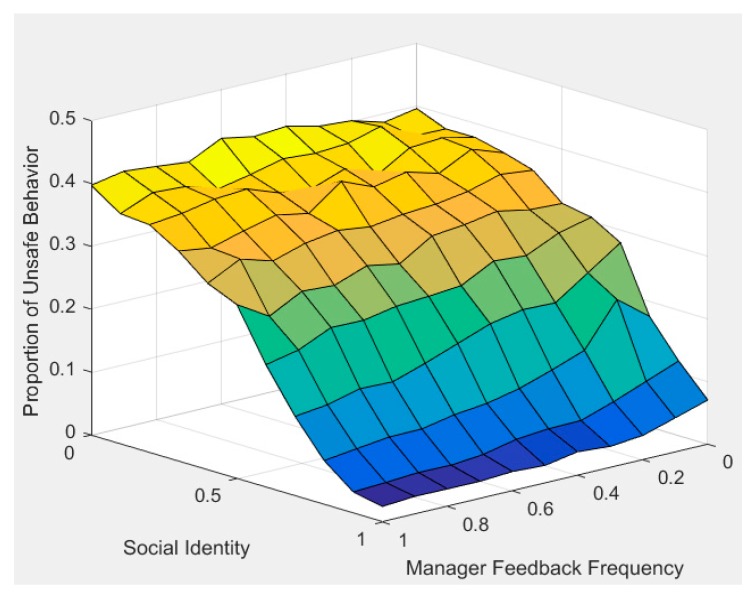
The influence of manager feedback frequency and social identity on workers’ unsafe behavior.

**Figure 11:**
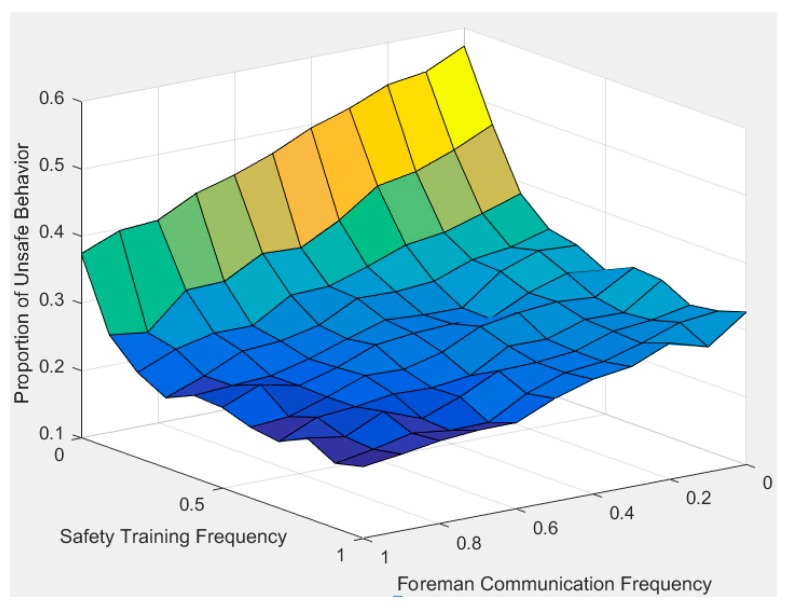
The influence of safety training frequency and foreman communication frequency on workers’ unsafe behavior.

Due to the multiple factors and dynamic characteristics of construction workers’ cognitive processes, two or more social organizational factors often change simultaneously in reality. Figure 10 and Figure 11 examine the impact of simultaneous changes of two social organizational factors on construction workers’ unsafe behavior.

Figure 10 shows the effect of manager feedback frequency and social identity on workers’ unsafe behavior. As shown in the figure, when workers receive high-frequency manager feedback (MF = 1) and their social identity is low (q = 0), the proportion of unsafe behavior is still high (unsafe behavior percentage = 40%). This shows that even if workers get positive feedback from managers, their low recognition of managers makes it difficult to achieve good safety performance. In contrast, when workers receive low-frequency manager feedback (MF = 0), but with high social identity (q = 1), the proportion of unsafe behavior is low (unsafe behavior percentage = 10%). Although workers receive low-frequency manager feedback, their high social identity may help them get feedback and communication from the foreman and managers, so the proportion of unsafe behavior is therefore lower. When the worker’s social identity and manager feedback frequency are both at the highest level (MF = 1, q = 1), the proportion of workers’ unsafe behavior is the lowest. The simulation results fully demonstrate the impact of social identity on workers’ unsafe behavior. Only when workers fully agree with the guidance of managers, will they effectively accept feedback from managers about their unsafe behavior.

Figure 11 shows the effect of manager feedback frequency and foreman communication frequency on workers’ unsafe behavior. As shown in the figure, when workers receive high-frequency safety training from managers ($CN_{i3}^{t}$ = 1) and keep a high frequency of safety communication with the foreman ($CN_{i2}^{t}$ = 1), the number of workers’ unsafe behaviors is smallest (unsafe behavior percentage = 20%). On the contrary, when workers receive low-frequency safety training from managers and keep a low frequency of safety communication with the foreman, the amount of unsafe behaviors is biggest (unsafe behavior percentage = 57%). Therefore, proper safety training and communication with the foreman are both necessary to effectively improve workers’ safety awareness and safety knowledge, thereby reducing workers’ unsafe behavior.

## 5. Discussion

This research develops an agent-based modeling approach to understand the sociocognitive process of construction workers’ unsafe behavior. Well-established theories (e.g., cognitive psychology [35] and social learning [82]), model validation test (e.g., [39,60,72]), and related research on managers, foreman, and coworkers (e.g., [23,24,41]) are used during the process. The model was established using MATLAB R2015b software (The MathWorks, Natick, MA, USA), relevant empirical data were used as initial values, and the validation of the model was tested. The model validation was also proven by the consistency between the baseline model and previous empirical studies. Regression analysis and Pearson correlation test were also used to ensure the qualitative consistency of the baseline model and the reality. Lastly, the risk acceptance and unsafe behavior changes of workers in interaction with managers, foremen, and coworkers were simulated, and the reasons for the failure of workers’ sociocognitive processes were explained. The impact of single-factor and two-factor management measures on workers’ unsafe behaviors was tested. The order of discussion corresponds with that of the results. Section 5.1 discusses the impact of social groups on unsafe behavior of construction workers. In Section 5.2, the cognitive causes of unsafe behavior of construction workers are discussed. While Section 5.3 to Section 5.5 discuss the impact of social organizational factors on workers’ unsafe behaviors, Section 5.6 and Section 5.7 discuss the practical significance and limitations of the developed model.

### 5.1. Implications of Social Group on Workers’ Unsafe Behavior

The simulation result shows that the coworker norm regarding safety tends to be no stricter than the manager norm, which is basically consistent with the research of Choi et al. [56]. Managers tend to have a lower level of risk acceptance than coworkers, so the manager norm is always stricter. Managers are responsible for workers’ safe production, and their safety norms are therefore relatively high. From the perspective of coworkers, their norm is often higher than the workers’ risk acceptance, which means the influence of coworkers has been argued to not always be positive [25,83,84]. Coworker norm includes many wrong norms, such as coworkers’ unsafe behaviors and incorrect safety concepts, which are also emulated and learned by workers. At the construction site, managers should fully make use of manager norms in safety and avoid workers’ emulation on coworkers’ unsafe behaviors.

When the foreman norm is excluded, workers’ unsafe behavior ratio is 13.50% higher than the baseline model, which is greater than the situation excluding manager norm or coworker norm. This fully demonstrates the important role of foreman in reducing workers’ unsafe behaviors. In construction, the foreman is the leader of the work crew, and the individual observes the crew on a daily basis [23]. The foreman is responsible for production and team safety. Strengthening the training of foremen in fall prevention and safety communication will significantly improve safety performance of workers [27]. In addition, the foreman has not only a management role but also a demonstration role for workers. When the foreman’s demonstration role is excluded, workers’ unsafe behavior percentage is 6.13% higher than the baseline model. This means that ignoring the demonstration role of the foremen will underestimate their role in site safety management. Although previous studies have made meaningful contributions to the management role of workers, the foreman’s demonstration role is poorly considered [23]. This paper is helpful to enrich the theory of social learning. Social learning theory implies that individual behavior is the result of the interaction of cognition, action, and environment [82]. In the construction industry, in addition to the behavior feedback of managers, the foreman’s demonstration role is still an effective way for workers to learn safety knowledge. Thus, both behavior feedback and the demonstration role should be included in the category of social learning. It is suggested that, in addition to oral safety knowledge exchange on the job site, foremen need to set an example and show workers more safety behaviors. For example, the foreman should use his own actions as a demonstration to show workers the correct method of wearing a helmet and the right way to tie a safety rope, so that workers may have deeper memories of the correct actions. In practice, managers should not only pay attention to the behavior of workers, but also pay attention to the behavior of the foremen. As the foremen’s behavior is regulated, workers will perform safer behavior.

### 5.2. Sociocognitive Causes of Unsafe Behavior of Construction Workers

This study also made a contribution to providing quantitative evidence to uncover the reasons for the failure of construction workers’ cognitive processes. In the simulation, workers’ *PR*, *UR*, *RA*, *UB,* and other parameters are expressed as quantitative values to analyze three causes of cognitive process failure of construction workers, including not finding risk, underestimating the risk, and correctly evaluating the risk but not performing safe behaviors. By analyzing the sociocognitive process of construction workers, managers can not only understand the stages of cognitive failure of construction workers but also understand the factors that affect the failure of cognitive stages. This mechanism also helps to identify the key factors and critical paths, facilitating the redevelopment of safety guidelines in the construction industry and also helps managers propose better management countermeasures for the failure of workers’ cognitive processes. 

Case 1 showed that the worker ultimately performed unsafe behavior because he did not find the danger. This situation is greatly related to the limitation of workers’ sense organs [15], such as sense failure caused by fatigue and unseen hazards [15]. Some external equipment can reduce worker fatigue. Kulkarni and Devalkar [85] pointed out that for activities like transportation of materials, trolleys or conveyor belts should be used to ease the lifting action and reduce external loads on the worker’s body. At the same time, workers do not realize that the danger may be caused by insufficient safety awareness and safety knowledge. Measures such as construction safety signs, safety information sharing, and establishment of a safety information platform can help construction workers fully grasp building safety information, increasing their safety awareness.

Case 2 indicated that the workers’ finding the risk without understanding it leads to unsafe behavior. This is also common in previous literature. According to the research of Lombardi, a worker chooses not to wear glasses when he needs to grind a piece of metal down quickly, just because “it takes 10 seconds” [22]. Obviously, the worker underestimates the dangers without glasses. The abundant safety knowledge and good safety attitude enable workers to correctly estimate risks. By introducing the safety card system and establishing safety training mechanisms, managers can enrich workers’ safety knowledge and avoid workers’ underestimating the risk. In addition, changes of attitude are expected to be effective for new workers [86]. By delivering and evaluating a simple behavioral attitude measurement form, managers can always understand the safety attitude of novice construction workers and provide them (especially those with unsafe attitudes) targeted education and training on a regular basis.

In Case 3, the worker correctly assessed the risk, but mistakenly thought that his risk tolerance was high enough and performed unsafe behavior. This is often associated with loose social norms. Under the loose coworker norm with low-risk acceptance, workers often behave as “tough guys” to avoid being laughed at by coworkers [21]. Stricter safety norms should be advocated. Managers should reward workers who have been behaving safely and encourage workers to learn more from coworkers’ safety behaviors in toolbox talk. 

Case 4 revealed a situation in which the worker underestimated the risk at the beginning, but stricter manager norm and foreman norm made the worker have lower risk acceptance levels and took safe behaviors. This means cognitive biases in one stage can be corrected under the social norm, which is supported by the findings of some previous research. The order workers repeatedly remind a forgetful young worker to wear goggles, helping him to former a good safety habit [22]. This means that experienced, older workers often have a strong sense of safety regulations and could urge workers to develop safer subjective norms. Cases 3 and 4 show that the worker forms his subjective norms through interaction with coworkers, foremen, and managers. Under looser safety norms, workers will tend to be unsafe. Conversely, under safety-oriented social norms, workers’ unsafe behaviors may be corrected. It can be explained by social psychology theory that human behavior, including mental activity, is the result of the individual interaction and is also influenced by a group environment [87]. However, most of the previous literature only focused on the mechanism of workers’ individual cognitive processes. Under the social cognitive mechanism, workers’ cognitive failure at one stage may be corrected and need to be further considered. This research therefore helps to improve the theory of cognitive psychology of construction workers. Consequently, managers should give full play to the role of social groups in correcting workers’ cognitive biases. When a worker has a cognitive bias at one stage, the manager should tell him/her about his/her mistake and correct it in a timely manner.

### 5.3. Effects of Social Identity on Safety Behavior

After examining the causes of the failure of the cognitive process of construction workers, this study simulated the impact of different management measures on workers’ unsafe behaviors. The findings that effects of social identity have the strongest influence on safety behavior suggest that improving workers’ social identity can significantly enhance their safety behavior, which can be explained by social identity theory. When a particular group identity is salient, people tend to try to conform to the salient group’s norm [88]. Normally, foremen and managers together form salient groups, and their risk acceptance is generally lower as compared with coworkers. After conforming to the safety norm of foremen and managers, workers’ safety norm is improved and unsafe behaviors are controlled. Previous literature provides effective ways to enhance workers’ social identity. Firstly, communication between group members and safety learning activities are proven to be capable of enhancing workers’ sense of belonging and team cohesion [89,90]. Secondly, managers’ use of inclusive language will promote identification with social identity. As Seyranian’s research shows, humor helps a team form a positive cultural cohesion [91]. Managers do not need to always keep a straight face to workers, and joking appropriately during the meeting is beneficial for improving social identity of workers. 

### 5.4. Effects of Safety Meeting and Safety Communication on Safety Behavior

Safety training and safety communication have a significant effect on safety performance, which echoes previous research [13,54,92,93]. More frequent safety training and foreman safety communication help workers have a stronger safety awareness and more safety knowledge. This, according to the above result, enhances workers’ risk perception and understanding of risks, thereby reducing their own unsafe behavior. Unfortunately, poor safety communication is a significant problem in the construction industry [13]. Borys [94] found that when safety hazards and injury prevention methods are identified and discussed during safety planning meetings, most workers are largely uninvolved. Thus, effective methods to improve safety communication and safety training should be proposed. Firstly, the government should play a promoting and leading role in safety training and safety communication. Thirty-four percent of the respondents in the study of Tam et al. [48] suggested that the government should organize security training, but the role of government is still limited. The government should actively formulate training outlines and strategies, and enforce them by law in order to ensure workers in both large and small businesses have the opportunity to receive adequate and well-structured safety training. Secondly, not only does safety training need to be provided to workers, but safety knowledge, communication, and effective leadership skills need to be provided to managers and foremen [27,95]. Thirdly, new technologies such as AR (Augmented Reality) and VR (Virtual Reality) technology can be adopted as a new way of safety training [96,97]. The advantages of using VR in education and training are related to its ability to enable students to interact with each other within virtual three-dimensional (3D) environments [96]. Using VR, AR, and other technologies to simulate the environment of the construction site allows workers to recognize the danger sources and various unsafe behaviors at the construction site, which will play a better role in safety training. At the same time, the results also show that safety training has a significant effect on reducing workers’ unsafe behavior when it starts to improve, and when it reaches a certain level, it reduces the worker’s unsafe behavior less significantly. The findings are in line with results of Zhang et al. [26]. Due to its marginal effect, excessive safety training is not necessary, so the combination of various forms, such as manager feedback, safety participation, and safety care is preferred and helps better reduce workers’ unsafe behaviors.

### 5.5. Effects of Behavior Feedback on Safety Behavior

Behavior feedback plays an important role in reducing workers’ unsafe behaviors, which has not only been found in the construction industry but also in other industries such as coal mines and papermaking [98,99]. The manager and foreman should always provide positive or negative feedback according to workers’ actions, and let them know the correctness of their behaviors to encourage more safe behaviors. Choudhry’s research shows that based on goal setting, feedback, and effective measures of safety behavior, it significantly improves safety performance at construction sites [100]. Therefore, behavior feedback needs to be combined with established goals. Before on-site work, the project manager should set specific safety goals and output targets for workers. After workers complete the construction tasks, the manager gives feedback to workers’ behavior, points out the gap between the current behavior and the established goals, and helps workers improve their safety performance. Chua et al. [101] pointed out that feedback should include two levels: first, feedback on failures, and second, feedback on future safety planning. At present, feedback from managers is often concentrated on the first level. When workers take unsafe behaviors, managers often point out that this is not safe and should not be done, but few managers make correct safety planning and safety guidance for workers in advance. Safety planning is a long-term guarantee mechanism for workers’ safety. Managers should make a safety feedback plan for each worker and make regular feedback on future safety plans. In addition, the feedback methods should be diverse, including not only behavior feedback, but also rewards and penalties in wages. Incentives are sometimes more effective than criticism. Therefore, both criticism and reward feedback mechanisms should be advocated.

### 5.6. The Practical Significance of the Model

The proposed model can be used to develop a series of management measures to reduce workers’ unsafe behaviors. For example, the initial value of baseline model is exemplary. In practice, the initial value can be adjusted according to the actual situation to predict the impact of management measures on workers’ unsafe behaviors. By analyzing single-factor management measures and multi-factor management measures, the optimal management strategy is determined. In addition, this paper quantifies the workers’ risk understanding, risk acceptance level, and other cognitive factors. Workers’ risk understanding and risk acceptance levels can be predicted in real time. When there is a deviation in risk understanding, a series of interventions such as safety training and safety communication is adopted to correct workers’ cognitive deviations. On the other hand, this model emphasizes the effect of foreman’s demonstration role on workers’ unsafe behavior. Practically, the foreman should use his/her own behavior as a demonstration to reduce workers’ unsafe behavior.

### 5.7. Limitation and Future Work

However, the proposed model bears considerable limitations that should be further discussed in future research. First, the initial value of the experiment was based on the empirical data and the author’s understanding of the construction site. It is still unknown whether it is applicable to different projects. For example, for projects with high risks, the frequency of training and communication may change, and the effects of various management measures on workers may be different. In future research, more data are expected to be obtained through questionnaires, ERP (event-related potential) experiments, etc. to test the accuracy of the model. Second, this research simplifies some relationships, and e.g., the physiological and psychological factors of workers (such as fatigue) are not covered (however, these may also be important factors affecting unsafe behavior of construction workers). However this study focuses on the impact of social organizational factors on the cognitive process of workers; rather than enumerating all the insecure factors that affect workers, proper simplification is therefore appropriate. This is justified because an appropriate level of model simplification and empirical realism is important, while an accurate representation of all the details of a construction project is not possible [102]. Other detailed influence paths are worth exploring with reference to sociology and psychology theories. Third, some management parameters in this paper are expressed by frequency (e.g., safety training frequency) without considering the impact of safety training intensity on workers’ unsafe behaviors. The impact of dual intensity and frequency management effects on worker behaviors can be explored in future research. Ultimately, this paper simulates workers’ unsafe behavior from the perspective of social relations at construction sites and does not design the simulation environment for a specific type of work. Future research can deepen understanding of building safety-related behaviors and sociocognitive process in a variety of complex simulation environments, and promote the design of safety management strategies in practice.

## 6. Conclusions

This study adopts an Agent-Based Modeling approach to study construction workers’ sociocognitive process with the interaction with coworkers, managers, and foremen. According to the knowledge of cognitive psychology and social psychology, the relationship between social organizational factors and workers’ individual cognitive factors were identified, and a sociocognitive model of construction workers’ unsafe behavior was constructed. The model confidence was built by implementing qualitative and quantitative agreement tests. A series of simulation experiments was subsequently conducted to explore the reasons for the failure of workers’ sociocognitive processes and the impact of different management measures. The simulation results show that there are differences in the impacts of three social groups on workers. Specifically, foremen’s demonstration role is often more effective in reducing workers’ unsafe behavior than safety communication and should therefore not be ignored. Manager’s behavior feedback and safety training have a huge impact on workers’ unsafe behavior, but excessive safety training is not effective. Coworkers’ demonstration role on workers’ safety behaviors is not always positive. These three types of social norms have an important impact on workers’ safety norms and often correct workers’ cognitive errors. Among various social organizational factors, social identity has the most obvious effect on reducing workers’ unsafe behaviors, and preventive measures are more effective than reactive measures in reducing workers’ unsafe behavior. Therefore, the findings from this research allow practitioners to proceed from the workers’ whole sociocognitive process, give full play to the effectiveness of social groups, and take appropriate management measures to reduce workers’ unsafe behaviors. This research also contributes to the application of the ABM approach to workers’ sociocognitive processes and site safety management. 

## Figures and Tables

**Table 1:** Initial parameter.

Parameter	Value
Time	280
Number of working groups	5
Number of workers per working group	20
Actual risk	Triangular (0.1,0.5,0.9)
Workers’ initial attitude	U(0.4,0.9)
Workers’ initial risk understanding coefficient	U(0.6,1.2)
Workers’ initial safety awareness	0.8
Workers’ initial safety knowledge	0.8
Workers’ social identity	0.5
Weight of coworker norm	0.2
Weight of foreman norm	0.45
Weight of manager norm	0.35
The frequency at which the foreman gives workers positive feedback	0.1
The frequency at which the foreman gives workers negative feedback	0.6
The frequency at which the manager gives workers positive feedback	0.1
The frequency at which the manager gives workers negative feedback	0.6
The frequency of manager safety training	0.5
Frequency of communication between workers and coworkers	0.3
Frequency of communication between workers and foreman	0.3
The improvement in worker safety knowledge by manager safety training	0.1
The effect of manager safety training on workers’ safety awareness	0.2
The effect of communicating with coworkers to improve workers’ safety awareness	0.01
The effect of communicating with foreman to improve workers’ safety awareness	0.2

**Table 2:** Pearson correlation test.

Influencing factors	Statistics	Safety Awareness	Safety Knowledge
Safety Communication	Pearson correlation	0.705 **	0.690 **
	Significant (bilateral)	0.000	0.000
	*N*	280	280
Safety Training	Pearson correlation	0.735 **	0.743 **
	Significant (bilateral)	0.000	0.000
	*N*	280	280

Note: ** *p* < 0.01

**Table 3:** Quantitative consistency between the simulation results and empirical data.

Items	Simulation Results	Empirical Data
Ratio of unsafe behavior	0.326	1/3 [11,79]
Ratio of near misses and accident	9.47:1	300:30 [39]
Rate of accident	3.35	3.2 [80]

**Table 4:** Workers’ unsafe behaviors ratio and accident rate (excluding the three social norms or the foreman’s demonstration role).

Types	Unsafe Behavior Ratio		Accident Rate	
	Value	Increased from Baseline Model	Value	Increased from Baseline Model
Excluding manager norm	0.346	6.13%	3.46%	3.28%
Excluding coworker norm	0.329	0.92%	3.36%	0.30%
Excluding foreman norm	0.370	13.50%	3.81%	12.07%
Excluding demonstration role of foreman	0.344	6.13%	3.41%	1.79%
Baseline Model	0.326		3.35%	

**Table 5:** The reasons for workers’ cognitive failure in different situations.

Parameter	Case 1	Case 2	Case 3	Case 4
*AR*	0.480	0.439	0.439	0.439
*PR*	0	0.522	0.453	0.595
*UR*	0	0.343	0.573	0.478
*RA*	0.716	0.475	0.629	0.403
*UB*	1	1	1	0
*AT*	0.931	0.656	0.844	0.578
*WN*	0.596	0.471	0.457	0.305
*FN*	0.676	0.258	0.342	0.237
*MN*	0.228	0.239	0.486	0.150
*ST*	0	0	0	1
*FCN*	0	0	1	0
*KL*	0.832	0.841	1.107	1.134
*SA*	0.832	0.841	1.007	1.034

Note: *AR* = actual risk, *PR* = risk perceiving, *UR* = risk understating, *RA* = risk acceptance, *UB* = unsafe behavior, *AT =* worker’s attitude, *WN* = coworker norm, *FN* = foreman norm, *MN* = manager norm, *ST* = safety training, *FCN* = foreman communication frequency, *KL* = worker’s safety knowledge, *SA* = worker’s safety awareness
